# Enhanced photocatalysis by coupling of anatase TiO_2_ film to triangular Ag nanoparticle island

**DOI:** 10.1186/1556-276X-7-239

**Published:** 2012-05-02

**Authors:** Jinxia Xu, Xiangheng Xiao, Feng Ren, Wei Wu, Zhigao Dai, Guangxu Cai, Shaofeng Zhang, Juan Zhou, Fei Mei, Changzhong Jiang

**Affiliations:** 1 Key Laboratory of Artificial Micro- and Nano-structures of Ministry of Education, Wuhan University, Wuhan, 430072, People ’ s Republic of China; 2 State Key Laboratory of Electronic Thin Films and Integrated Devices, University of Electronic Science and Technology of China, Chengdu, 610054, People ’ s Republic of China; 3 Center for Electron Microscopy and School of Physics and Technology, Wuhan University, Wuhan, 430072, People ’ s Republic of China; 4 School of Electrical & Electronic Engineering, Hubei University of Technology, Wuhan, 430068, People ’ s Republic of China

**Keywords:** plasmon, photocatalysis, nanospheres lithography, Ag nanoparticle island

## Abstract

In order to overcome the low utilization ratio of solar light and high electron-hole pair recombination rate of TiO_2_, the triangular Ag nanoparticle island is covered on the surface of the TiO_2_ thin film. Enhancement of the photocatalytic activity of the Ag/TiO_2_ nanocomposite system is observed. The increase of electron-hole pair generation is caused by the enhanced near-field amplitudes of localized surface plasmon of the Ag nanoparticles. The efficiently suppressed recombination of electron-hole pair caused by the metal-semiconductor contact can also enhance the photocatalytic activity of the TiO_2_ film.

## Background

TiO_2_, as a key photocatalyst, has received extensive attention during the past decades due to its strong catalytic activity, high chemical stability, nontoxicity, and low cost [1-5]. However, owing to its wide bandgap of 3.2 eV, only approximately 4% solar spectrum can be utilized and the conversion of photon to electron-hole pair is low. Furthermore, the high rate of electron-hole pair recombination limits the efficiency of photocatalytic activity. Therefore, how to enhance photocatalytic efficiency is very important for the widespread application of TiO_2_ as a photocatalyst. Recently, surface plasmon-mediated photocatalytic activity of TiO_2_ has become a hot research topic [6-9]. Surface plasmon resonance is produced by metal nanoparticles (NPs) due to photo-induced collective oscillation of conduction electrons on the surface of metal NPs when their size are smaller than the wavelength of the incident light beam (i.e., localized surface plasmon resonance, (LSPR)) [10]. The bandgap of TiO_2_ is 3.2 eV; near UV light (irradiation) can excite electron-hole pairs [11]. Ag NPs also show a very intense localized surface plasmon absorption in the near-UV region [12], which greatly enhances the electric field intensity in the vicinity of the Ag NPs. This enhanced near field at near-UV region could increase the light absorption to boost the excitation of electron-hole pairs in TiO_2_ and thus increase the efficiency of photocatalysis. This clearly indicates that the LSPR effect is a potential way for the enhancement of photocatalysis. The local field can be greatly enhanced in the vicinity of the triangular Ag NP array due to its unique morphology with sharp corners and edges. Theoretical studies on metal structures showed that the local field could be enhanced by several orders of magnitude in the NPs with special shape (e.g., triangular plates) [13]. Yang et al. [14] demonstrated that the LSPR of triangular Ag NPs was much stronger than that of sphere Ag NPs. In this study, we can get triangular Ag NP island on the surface of TiO_2_ film. The Ag/TiO_2_ composite film shows higher photocatalytic activity than the pure TiO_2_.

## Experimental section

**Figure 1:**
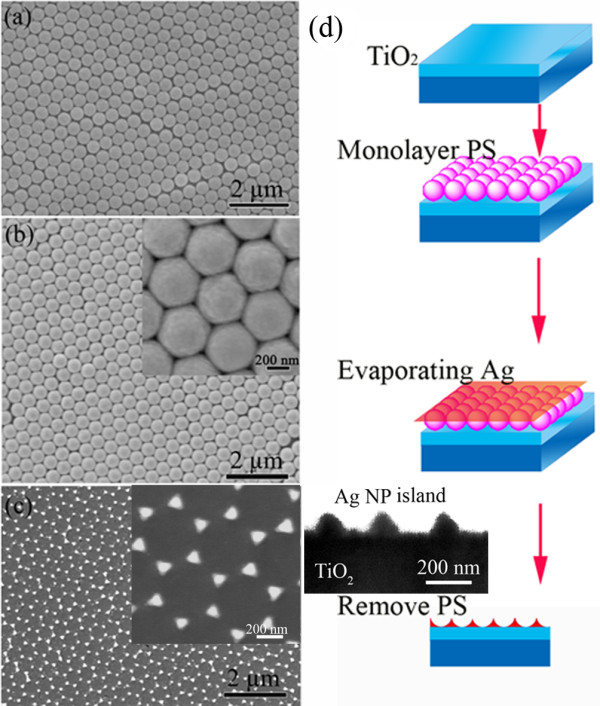
**(a) SEM image of the PS colloidal crystal template; (b) SEM image of deposited Ag film coated PS spheres monolayer.** The inset one is the magnification SEM image of (b); **(c)** SEM image of Ag NP island produced after removing the PS spheres monolayer; the inset one is a specially magnified Ag NP on the TiO_2_ film; **(d)** Schematic figure depicting the preparation of triangular metal NP island connected with semiconductor by PS lithography strategy, and the inset one is the cross sectional TEM image of Ag/TiO_2_ nanocomposite.

TiO_2_ film with a thickness of about 680 nm was prepared by direct-current reactive magnetron sputtering deposition on silica glass and then was subsequently capped with triangular Ag NPs (schematically described in Figure 1d). Well-ordered latex sphere template of polystyrene (PS) nanospheres (460 nm in diameter) was prepared on the TiO_2_ surface [15,16]. Metal Ag was deposited on the TiO_2_-PS surface by electron beam evaporation; the thickness of Ag film is approximately 100 nm. After the deposition of metal Ag, the PS nanospheres were removed by sonicating the sample in chloroform (CHCl_3_) for 20 s.

The structure of TiO_2_ film was investigated by grazing incidence X-ray diffraction (XRD). The morphologies of the self-assembled PS nanosphere mask and metal NPs were characterized by scanning electron microscopy (SEM) (FEI Sirion FEG, FEI Company, Eindhoven, The Netherlands). The microstructure of the samples was investigated using a JEOL JEM 2010 HT (JEOL Ltd., Akishima, Tokyo, Japan) transmission electron microscope (TEM) operated at 200 kV. Raman scattering spectra of all the samples were collected using a micro-Raman system. An Ar laser (488.0 nm) was used as the excitation source, and the laser power was kept at 10 mW.

The photocatalytic efficiency of TiO_2_ and Ag/TiO_2_ films with an area of 4 cm^2^ was evaluated by measuring the degradation rates of 5 mg/L methylene blue (MB) solution under UV irradiation. A mercury lamp (OSRAM AG, M ü nchen, Germany; 250 W with characteristic wavelength at 365 nm) was used as light source. Before irradiation, the samples were put in 40-mL MB for 30 min in darkness to reach absorption equilibrium. The decolorization of the MB solution was measured by a UV-vis spectrometer at the wavelength of 664.0 nm. The absorption spectrum of the MB solution was measured at a time interval of 30 min, and the total irradiation time was 4 h.

## Results and discussion

**Figure 2:**
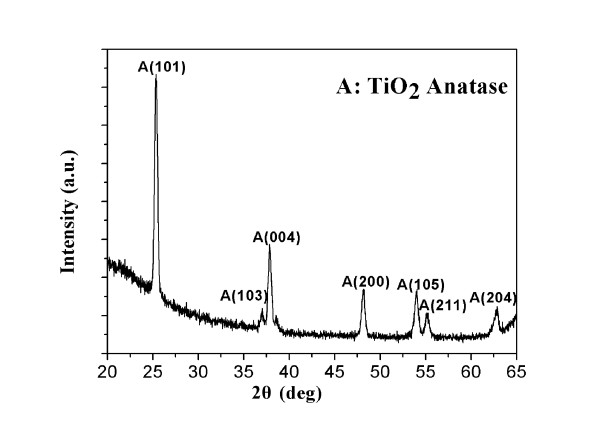
**XRD pattern of the TiO**_**2**_**film annealed at 500°C for 2 h in oxygen atmosphere.**

XRD was used to investigate the crystal phase of the TiO_2_ films after annealing at 500°C for 2 h in oxygen atmosphere. As shown in Figure 2, it can be seen that the annealed TiO_2_ film shows a typical anatase structure without any other detectable phase (JCPDS no. 21-1272), indicating the formation of anatase TiO_2_ nanocrystals [17].

Figure 1a shows the self-assembled monolayer arrays of nanospheres with a typically ordered hexagonal pattern on the surface of the TiO_2_ film. The SEM image of the PS nanospheres coated with Ag film deposited by electron-beam evaporation is shown in Figure 1b, and the inset is the magnification SEM image of the same sample. It demonstrates that the Ag film wraps the PS spheres uniformly and tightly. The morphology of the Ag NPs exhibits ordered hexagonal periodic arrays formed on the surface of the TiO_2_ film with a large area after removing the PS sphere masks, as shown in Figure 1c. The Ag NP island is triangular due to the shape of the interstitial voids in the shadow mask. The inset in Figure 1c shows the magnified SEM image of Ag NPs on the TiO_2_ film. The formation of Ag NPs on the surface of TiO_2_ film can also be observed in the cross-sectional TEM image of the Ag/TiO_2_ nanocomposite film in the inset of Figure 1d. Thus, the triangular Ag NP island with a large uniform area can be obtained by this method.

**Figure 3:**
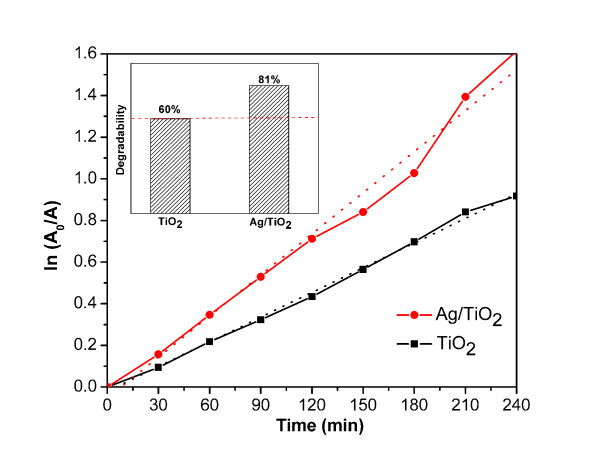
**Performances of Ag/TiO**_**2**_**nanocomposite film and TiO**_**2**_**film for photocatalytic degradation of MB under UV light irradiation.**

In order to verify the influence of the Ag NP island on the photocatalytic activity of the TiO_2_ film, the photocatalytic activity of Ag/TiO_2_ composite system was evaluated by degradation of the MB solution under UV irradiation at room temperature. For comparison, the pure TiO_2_ film was carried out under the same experimental conditions. As shown in Figure 3 (inset), the Ag/TiO_2_ composite system obtained higher photocatalytic efficiency (81%) than the pure TiO_2_ film (60%). Meanwhile, the photodegradation of MB can be assumed to follow the classical Langmuir-Hinshelwood kinetics [18], and its kinetics may be expressed as follows:

(1) $$\ln(\frac{A_{0}}{A})=kt$$

Where *k* is the apparent first-order reaction rate constant (min^-1^), *A*_0_ and *A* represent the absorbance before and after irradiation for time *t*, respectively. From the plots of $\ln(\frac{A_{0}}{A})$ versus the irradiation time shown in Figure 3, the *k* values obtained from the slops of the simulated straight line are 3.875  ×  10^-3^ and 6.372  ×  10^-3^ min^-1^ for the pure TiO_2_ film and Ag/TiO_2_ system, respectively. The rate of MB decomposition for the Ag/TiO_2_ nanocomposite is more than 1.6-fold as fast as that of pure TiO_2_ film. The results indicate that the Ag/TiO_2_ composite system exhibits better photocatalytic performance than the pure TiO_2_ film.

The electronic structure of TiO_2_ plays a key role in TiO_2_ photocatalysis. The increasing number of electron-hole pairs and the separation of electron-hole pairs at the surface of TiO_2_ are the key factors to improve the photocatalytic abilities of TiO_2_. Based on our experimental results and literatures, the photocatalytic activity enhancement could be explained as follows.

**Figure 4:**
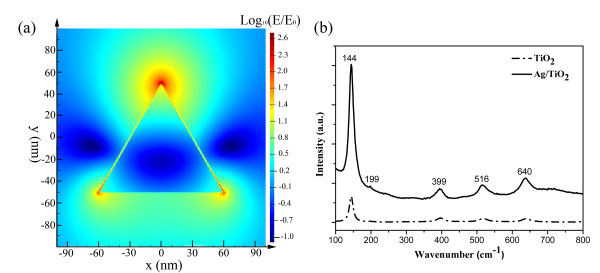
**(a) The local field intensity enhancement distribution E/E**_**0**_**in the logarithmic scale due to the presence of the equilateral triangular Ag NP island with 120 nm edge lengths and the incident wavelength is 480 nm.(b)** The Raman scattering spectra of pure TiO_2_ film and Ag/TiO_2_ nanocomposite film.

Firstly, the LSPR can be enhanced at the corner of the triangular Ag NPs, the incident light field coupling to the LSPR might induce the enhancement of absorption of light, which boosts the excitation of electron-hole pairs in TiO_2_, and therefore increase the efficiency of photocatalysis. The local field intensity enhancement distribution E/E_0_ in the logarithmic scale due to the presence of Ag NPs was simulated using the finite-difference time-domain method, as shown in Figure 4a. In this method, we assume that 80-nm-thick Ag equilateral triangular NPs with 120-nm edge length are arranged in a hexagonal lattice and that the incident wavelength is 480 nm. For simplicity in simulation, we assume that the Ag NPs are isolated from each other. In our structure, we consider *z* as the light incident direction, and *x* is the polarization direction; the highest enhancement is close to 10^3^ at the tip of the triangular Ag NP island. In order to prove the existence of the strong localized electric field induced by the triangular Ag NPs experimentally, we carried out a surface Raman scattering study. Figure 4b shows the Raman scattering spectra of the pure TiO_2_ film and the Ag/TiO_2_ nanocomposite film. All the samples observed in the Raman bands at 144, 199, 399, 516, and 640 cm^ − 1^ can be assigned to the Eg, Eg, B1g, A1g or B1g, and an overtone Eg vibration mode, respectively [19]. It is interesting to note that the Raman scattering is greatly enhanced in the Ag/TiO_2_ nanocomposite system compared with that in the pure TiO_2_ film. It is well known that Raman scattering intensity is proportional to the electric field intensity [20]. The stronger Raman scattering attained from the Ag/TiO_2_ nanocomposite indicates that a stronger electric field was induced by introducing the Ag NPs, which is consistent with the simulated results. Theoretical and experimental results both show a strong local electric field induced by the Ag NP island. This enhanced local electric field induced by the Ag NPs will be used for the photoactivity enhancement of the TiO_2_ film.

**Figure 5:**
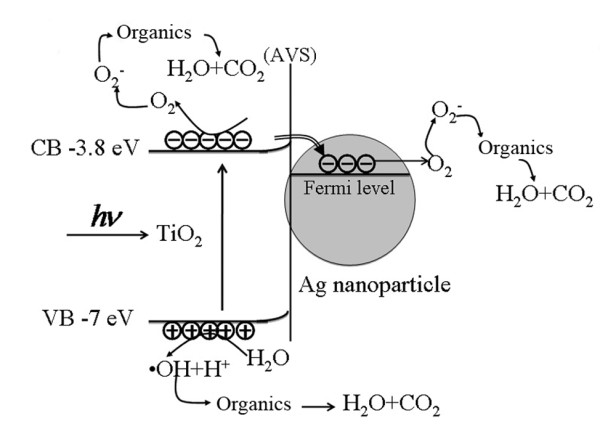
**Schematic diagram for the photodegradation of MB by Ag/TiO**_**2**_**nanocomposite system.**

Secondly, in the case of the Ag/TiO_2_ system, because of the lower work function of TiO_2_ (4.2 eV [21,22]) compared with that of Ag (4.52 to 4.74 eV [23]), theirs is a Schottky contact and will result in the upward bending of the energy band near the contact region. When TiO_2_ is excited by the incident light, electrons will move to the surface and accumulate there, while photo-generated holes remain in the TiO_2_. However, considering the work function of Ag which is higher than that of TiO_2_, the photon-generated electrons will transfer from TiO_2_ to Ag NPs (as shown in Figure 5); the electrons and holes will separate at the surface region. Moreover, there are fewer electrons transferring from Ag to TiO_2_ due to the Schottky barrier. The accumulated electrons at Ag NPs could be transferred to the oxygen absorbed on the surface to form superoxide ${\text{O}}_{2}{}^{-}$ or ${\text{O}}_{2}{}^{2-}$[24]. Accumulation of holes at the valence band of TiO_2_ leads to the production of surface hydroxyl radical ·OH [25], which is responsible for the oxidation decomposition of MB organics. Organic compounds are completely decomposed into water and carbon dioxide by reacting with the produced hydroxyl radicals [26]. Thus, the Ag/TiO_2_ composite system can efficiently suppress the recombination of photo-generated carriers and enhance photocatalytic activity.

## Conclusions

In conclusion, a highly ordered triangular Ag NP island on the surface of anatase TiO_2_ film was successfully prepared using the PS nanosphere lithography strategy. The Ag/TiO_2_ nanocomposite system can efficiently enhance photocatalytic activity. In addition, the Ag NP island will be of great significance to future applications in the fields of metal-semiconductor nanocomposite system photocatalysis for light-energy conversion.

## Competing interests

The authors declare that they have no competing interests.

## Authors ’ contributions

JX participated in material preparation, data analysis, and drafted the manuscript. XX conceived and co-wrote the paper. FR, WW, ZD, GC, SZ, JZ, and FM participated in the sample characterization. CJ participated in its design and coordination. All authors read and approved the final manuscript.
